# Incidence Rate of Hypersensitivity Reactions to Bee-Venom Acupuncture

**DOI:** 10.3389/fphar.2020.545555

**Published:** 2020-10-07

**Authors:** Eun-Jung Lee, Yo-Chan Ahn, Young-Il Kim, Min-Seok Oh, Yang-Chun Park, Chang-Gue Son

**Affiliations:** ^1^Department of Korean Rehabilitation Medicine, College of Korean Medicine, Daejeon University, Daejeon, South Korea; ^2^Department of Health Service Management, Daejeon University, Daejeon, South Korea; ^3^Department of Acupuncture and Moxibustion Medicine, College of Korean Medicine, Daejeon University, Daejeon, South Korea; ^4^Division of Respiratory System, Department of Internal Medicine, College of Korean Medicine, Daejeon University, Daejeon, South Korea; ^5^Department of Gastrobilliary System, Dunsan Hospital of Daejeon University, Daejeon, South Korea

**Keywords:** bee venom, adverse drug reaction, hypersensitivity, anaphylaxis, side effect, acupuncture

## Abstract

**Introduction:**

Bee-venom acupuncture (BVA) has been widely applied to various disorders including pain-related diseases; however, patients are often warned of adverse reactions such as anaphylaxis. This study aimed to estimate the risk of hypersensitivity reactions to BVA and to determine their clinical features.

**Methods:**

We retrospectively surveyed the medical records of patients treated by BVA between January 2010 and April 2019 in Dunsan Hospital of Daejeon University, and all cases of allergic reactions and their clinical symptoms were analyzed.

**Results:**

A total of 8,580 patients (males 4,081 and females 4,499) were treated with BVA which amounts to a total of 60,654 treatments (average 7.1 ± 14.8 times). A total of fifteen patients (7 males and 8 females) reported an allergic reaction (0.175%, 95% CI, 0.086–0.263) of type 1 hypersensitivity, indicating a rate of allergic reaction in 0.025% (95% CI, 0.012–0.037) of the total BVA treatments. The average number of BVA treatments in those patients was 6.9 ± 6.5 (males: 4.1 ± 3.4 and females: 9.3 ± 7.9). Among the cases of hypersensitivity reactions, 4 involved anaphylactic shock; therefore, the incidence rate of anaphylaxis was 0.047% (95% CI, 0.001–0.092) for the 8,580 subjects and 0.007% (95% CI, 0.000–0.013) for the 60,654 treatments. All grade 1 cases were recovered within 1 day, whereas others took up to 30 days for complete recovery.

**Conclusion:**

Our results may emphasize paying attention to unforeseeable risks of anaphylaxis after bee-venom acupuncture. This study could be essential reference data for the guidelines of appropriate use of bee-venom acupuncture and bee-venom-derived interventions in clinical applications.

## Introduction

Recently, venoms have received a lot of attention as an intervention for diseases. Among these, bee-venom is most commonly used as painkillers and anti-inflammatory drugs (Seo et al., 2017; Memariani et al., 2019). Furthermore, bee-venom is an effective therapeutic for other challenging disorders, including incurable skin disease, cancer, and Parkinson’s disease (Hartmann et al., 2016; Gu et al., 2018; Aufschnaiter et al., 2020). Despite these potential applications, however, the clinical use of bee-venom is limited due to allergic reactions, including life-threatening responses such as anaphylaxis.

In general, adverse drug reactions (ADRs) are one of the major reasons why the drugs were withdrawn from the post marketing phase (Demoly et al., 2008; Onakpoya et al., 2019). Drug hypersensitivity reactions account for 15% of ADRs (Pichler and Hausmann, 2016). Drug-induced anaphylaxis is the most serious and life-threatening hypersensitivity reaction (Turner et al., 2017) and many drugs that trigger anaphylaxis involving antibiotics, radiocontrast agents, and nonsteroidal anti-inflammatory drugs (NSAIDs) are frequently problematic in clinic (Wood et al., 2014; Giavina-Bianchi et al., 2018). Regarding NSAIDs-related incidence rate of hypersensitivity and anaphylaxis, one US clinical study reported as 0.30 and 0.02%, respectively (Blumenthal et al., 2017). Another study presented incidence rates of 0.48% for hypersensitivity reactions and 0.01% for anaphylaxis from 9,528 MRI examinations with contrast-agent (Li et al., 2006).

Bee-venom acupuncture (BVA) is a common therapeutic used worldwide, especially in Korean and Chinese clinics. Since one death by BVA anaphylactic shock was reported, the safety of bee-venom has been an important issue in Korea (Jung et al., 2012b). Two studies demonstrated partially BVA-related hypersensitivity reactions using data from clinical trials or hospital records (Park et al., 2015; Kim et al., 2016); however, further studies are still required to provide better clinical guidance for safe use of BVA. The present study aimed to estimate the risk size of hypersensitivity reactions by BVA and to determine their clinical features.

## Materials And Methods

### Study Design

This study was a retrospective analysis. The data source was the electronic medical records (EMRs) of 66,614 subjects who had visited the pain-spine center and rehabilitation center in Dunsan Hospital of Daejeon University, South Korea, from January 2010 to March 2019. This process was conducted *via* integrated hospital information system (IHIS) that analyzed the order communication system (OCS) and EMR. We first selected only subjects treated with BVA and then searched the cases with hypersensitivity after BVA treatment. We analyzed the incidence rate of hypersensitivity reactions and anaphylactic shock along with their clinical features. The inclusion criteria for the selection of patients and the incidence of a hypersensitivity reaction were as follows: 1) subjects who had been treated with BVA at least once, as recorded in their EMRs; or 2) subjects who complained of a BVA-related systemic allergic reaction with/without the prescription of anti-allergic drugs such as antihistamines, adrenaline, or adrenal cortex hormones. Strategy to identify the hypersensitivity cases was based on the guideline for BVA treatment, which all the cases of BVA allergic reaction should be referred to one conventional doctor in same hospital. We carefully reviewed the full medical records for all the suspected cases.

### BVA

The bee-venom source for BVA used in two centers was produced by Green Myeongpum Pharm Company (Namyangju, Korea). This bee-venom source was validated for its main active compound (over 99.9% melittin) and allergen-free purification (phospholipase A2, apamin, hyaluronidase, and histamine) and was then diluted to 10% with normal saline by Jaseng Namyangju Industrial Institute (Namyangju, Korea) or Kirin Korean Medicine Industrial Institute (Wonju, Korea). BVA treatment was conducted with an injection of bee-venom solution (range, 1.0 to 2.0 mL) at a specific acupoint of skin or muscle with interval of 3 or 4 days.

### Ethics Statement

This study was approved by the Institutional Review Board for Human Research of Daejeon University Dunsan Hospital (approval number: DJDSKH-18-E-08-2). We have obtained verbal consent from every subject identified as having a hypersensitivity reaction.

### Data Source Extraction and Analysis

We extracted general information on the subjects treated with BVA including gender, age, frequency of BVA prescriptions and the main health complaint (according to the International Classification of Diseases-10; ICD 10) and cases of hypersensitivity reaction including its primary symptoms, the recovery period, and the presence of a family history of allergy-related illness. We, thus, calculated the incidence rate of hypersensitivity reactions and anaphylactic shock, with the number of subjects and times of BVA treatment. The type of hypersensitivity reaction was classified according to immune mechanisms and clinical symptoms (Dispenza, 2019). The severity of the allergic symptoms was assessed in three categories proposed by Brown SG (Brown, 2004), which was established by slight modification from Muller’s four classifications (Mueller, 1966), while the definition of anaphylaxis followed the 2010 World Allergy Organization guideline (Simons et al., 2011) as shown in **Supplementary Table 1**. For every subject identified as having a hypersensitivity reaction, we conducted a telephone survey to clarify the consequences and other medical events since they stopped BVA treatment.

### Statistical Analysis

Categorical variables were expressed as frequency and percentages, and averages were expressed as the means ± standard deviation (SD). The incidence rate was given as a percentage within the 95% confidence interval. The comparison between male and female was analyzed using chi-squared test or Fisher exact test. Statistical analyses were performed with the SPSS statistical software package version 18.0 (SPSS Inc., Chicago, IL, USA).

## Results

### Characteristics of Subjects

From EMR analysis, 8,580 subjects (4,081 males and 4,499 females) met our study criteria. The total number of BVA prescriptions was 60,654, accounting for an average of 7.1 ± 14.8 BVA treatments per subject (males: 7.0 ± 11.8 vs. females: 7.1 ± 17.1, p > 0.05). The median age of the subjects was 53 years (range, 13–98 years). Most subjects (93%) had complaints of the musculoskeletal system and connective tissue or injury-related pain. The number of subjects were counted without duplication, no matter how many times they visited hospitals as an inpatient or outpatient (**Table 1**).

**Table 1:** Characteristics of subjects.

Subjects	Male/Female (%)	Total
Number of subjects (%)	4,081 (47.6)/4,499 (52.4)	8,580 (100)
Median age (year, range)	49 (13 to 88)/53 (13 to 98)	53 (13 to 98)
Number of BVA treatments (%)	28,626 (47.6)/32,028 (52.4)	60,654 (100)
Mean N. of BVA treatments	7.0 ± 11.8/7.1 ± 17.1	7.1 ± 14.8
**Main complains (ICD-10)**		**N. of subjects (%)**
M00-M99: Diseases of the musculoskeletal system and connective tissue		4,724 (50.2)
S00-T98: Injury, poisoning and certain other consequences of external causes		4,027 (42.8)
G00-G99: Diseases of the nervous system		449 (4.8)
R00-R99: Symptoms, signs and abnormal clinical and laboratory findings, NEC		95 (1.0)
Others		110 (1.8)
Total subjects*		9,405 (100)

*Duplication was permitted to be counted in case one patient received BVA treatment as an inpatient and an outpatient. Therefore, this number is larger than number for analysis (8,580 subjects).

### Incidence Rates of Hypersensitivity Reaction and Anaphylaxis

Fifteen subjects (seven males and eight females) showed hypersensitivity reactions to BVA, with an incidence rate of 0.175% (95% CI: 0.086–0.263%) from 8,580 subjects and 0.025% (95% CI: 0.012–0.037%) from 60,654 treatments. Among those, four cases were anaphylactic shock, corresponding to 0.047% (95% CI: 0.001–0.092) of subjects and 0.007% (95% CI: 0.000–0.013) of treatments, respectively. No significant difference by gender was observed for total hypersensitivity. The anaphylactic shock was more frequent in female than male for both subjects (0.067% in female vs. 0.025% in male) and treatments (0.003% in female vs. 0.009% in male), but no statistical significance was observed (p > 0.05, **Table 2**).

**Table 2:** Incidence rate of hypersensitivity reaction and anaphylactic shock.

Hypersensitivity reaction	Incidence rate (95% CI)
By subjects (15 of 8,580)Male (7 of 4,081)Female (8 of 4,499)	0.175% (0.086–0.263)0.172% (0.045–0.298)0.178% (0.055–0.301)
By treatments (15 of 60,654)Male (7 of 28,626)Female (8 of 32,028)	0.025% (0.012–0.037)0.024% (0.006–0.043)0.025% (0.008–0.042)
**Anaphylaxis shock**	**Incidence rate (95% CI)**
By subjects (4 of 8,580) Male (1 of 4,081)Female (3 of 4,49	0.047% (0.001–0.092) 0.025% (-0.024–0.073) 0.067% (-0.009–0.142)
By treatments (4 of 60,654) Male (1 of 28,626) Female (3 of 32,028)	0.007% (0.000–0.013) 0.003% (-0.003–0.010) 0.009% (-0.001–0.020)
**Severity of symptom**	**Frequency (%)**
Grade 1 (Mild)Grade 2 (Moderate)Grade 3 (Severe)	6 (male 4, female 2), 40.0%7 (male 3, female 4), 46.7%2 (male 0, female 2), 13.3%

### Clinical Features of Hypersensitivity Reaction and Anaphylaxis

Regarding the severity of the symptom, 6 cases were grade 1, while 7 and 2 cases were belonged to grade 2 and 3, respectively (**Table 2**). All cases of hypersensitivities appeared immediately or within one hour after BVA treatment, which is the typical feature of type 1 hypersensitivity. They completely recovered, although one subject was hospitalized. Skin symptoms such as generalized itchiness and urticaria were the most common (80%), followed by respiratory symptoms (46.7%), cardiovascular and neurological symptoms (26.7%), and gastrointestinal symptoms (20%). Twelve out of 15 cases occurred after several BVA treatments, while three cases showed the hypersensitivity reactions on the first exposure in our hospital (**Table 3**).

**Table 3:** Characteristics of 15 allergic reaction cases with bee-venom acupuncture.

Case (sex/age)	Chief complaints (allergy disorders^*^)	Family history of allergy	No. BVA treatment^†^ (Interval^‡^)	Allergic reaction		
				Main symptoms	Type (Grade)^§^	Outcome (follow-up)^#^
P1 (M/36)	Cervical sprain	Fish allergy (mother)	1 (0 d)	Urticaria	Type1 (G 1)	Recovered (1d, itch)
P2 (M/38)	Cervical sprain	None	2 (14 d)	Urticaria	Type1 (G 1)	Recovered (1d, itch)
P3 (M/44)	Cervical sprain	Asthma (brother)	4 (3 d)	Urticaria	Type1 (G 1)	Recovered (1d, itch)
P4 (M/48)	Lumbar HNP	None	3 (7 d)	Urticaria	Type1 (G 1)	Recovered (1d, itch)
P5 (F/53)	Cervical HNP (aspirin allergy)	None	1 (0 d)	Urticaria	Type1 (G 1)	Recovered (1 d, itch)
P6 (F/56)	Cervical sprain	None	1 (0 d)	Urticaria	Type1 (G 1)	Recovered (1 d, itch)
P7 (M/32)	Knee contusion (allergic rhinitis)	Allergic rhinitis (brother)	2 (8 d)	Dizziness, throat tightness, weakness, anxiety	Type1 (G 2)	Recovered (1d, dizziness)
P8 (M/64)	Sprain of knee	None	11 (22 d)	Urticaria, constriction in chest, weakness	Type1 (G 2)	Recovered (30 d, weakness)
P9 (F/32)	Cervical sprain	None	14 (64 d)	Urticaria, localized edema, throat tightness	Type1 (G 2)	Recovered (2 d, itch)
P10 (F/42)	Cervical sprain (allergic rhinitis)	None	22 (7 d)	Headache, dizziness, numbness, abdominal pain, generalized pain	Type1 (G 2)	Recovered (21 d, dizziness)
P11 (F/42)	Frozen shoulder (contrast agent allergy)	None	15 (4 d)	Urticaria, erythema, throat swelling	Type1 (G 2)	Recovered (3 d, throat swelling)
P12 (M/70)	Lumbar stenosis (asthma)	None	6 (362 d)	Urticaria, pain, chilling, cold sweat, nausea, vomiting, anxiety, dyspnea	Type1 (G 2)	Recovered (30 d, anxiety)
P13 (F/62)	Lumbar HNP	None	13 (231 d)	Urticaria, paresthesia, throat tightness, dyspnea	Type1 (G 2)	Recovered (2 d, weakness)
P14 (F/59)	Spinal stenosis	None	2 (391 d)	Urticaria, throat tightness, chest pain, nausea, vomiting, dizziness, dyspnea, hypotension, loss of consciousness	Type1 (G 3)	Recovered (14 d, dizziness, weakness)
P15 (F/60)	Cervical HNP (allergic rhinitis)	Allergic rhinitis (son)	6 (129 d)	Weakness, paresthesia, throat tightness, dyspnea, lip edema, hypotension, loss of consciousness	Type1 (G 3)	Recovered (3 d, lip edema)

*Presence of allergy disorders in an ordinary day. ^†^Number of BVA treatments by the day of an allergic reaction. ^‡^Duration between two time points (the last BVA-treatment and the next previous BVA administration). ^§^Severity of allergic reactions according to the grading system. Grade 1, 2, and 3 refer to ‘mild’, ‘moderate’, and ‘severe’ for classification of symptom’s severity. ^#^The time point when the last symptom(s) disappeared.

## Discussion

In this study, we investigated the clinical question: what is the risk size of BVA-derived hypersensitivity reactions, including anaphylactic shock and its clinical features. Most of the subjects had disorders related to the musculoskeletal system and joints, on which bee-venom and its main components may have a therapeutic effect as a painkiller or chondroprotective agent (Jeong et al., 2015). The incidence rate of hypersensitivity reactions in our study (0.175% from 8,580 subjects and 0.025% from 60,654 treatments) was slightly lower than results from another retrospective study, which showed a 0.23% incidence rate of BVA-induced hypersensitivity reactions (Kim et al., 2016). Drug-induced hypersensitivities can be broadly classified into four types by immune mechanisms (Dispenza, 2019). BV-induced allergic reaction is known mainly to be of type 1 hypersensitivity which is mediated by IgE (Kwon et al., 2009).

In our results, all cases with hypersensitivities appeared immediately or within one hour after BVA treatment, indicating the typical feature of type 1 hypersensitivity. Their clinical severities were 40.0%, 46.7%, and 13.3% for grade 1 to grade 3, respectively (**Table 2**). In general, anaphylaxis is the most severe systemic hypersensitivity reaction. In the United States, 1.6–5.1% of the population are estimated to have experienced anaphylaxis, and 1% of hospitalizations for anaphylaxis showed fatal results (Ma et al., 2014; Wood et al., 2014). The contrast agents for MRI examination are well known to show a high rate of anaphylactic reactions. Our data showed four cases of anaphylaxis due to BVA, corresponding to 0.047% of subjects and 0.007% of treatments (**Table 2**), which is very similar to the overall incidence rates of MRI contrast agents. One Korean group analyzed MRI contrast agent-related adverse reactions according to both patients (84,367 patients) and doses (141,623 total doses) as we did, presenting 0.121% (patients) and 0.079% (doses) for hypersensitivity and 0.01% (patients) and 0.008% (doses) for anaphylaxis (Jung et al, 2012a). Although our study and other studies showed the two kinds of the incidence rate of hypersensitivity reactions and anaphylaxis (patients vs. treatments), treatment-derived rates would be underestimated due to the repeated BVA treatments likely 5 to 10 BVA sessions per patient. Accordingly, we have to notice that the patient-derived rates reflect further practically the risk sizes comparing with the treatment-derived rates.

In general, the occurrence of drug-related adverse reactions, including anaphylaxis, depends on the three main factors: the drug properties themselves, the genetic backgrounds of subjects and environmental factors such as the coadministration of drugs, alcohol or food, underlying disorders, gender, age, or even psychiatric status (Merle et al., 2005; Nguyen et al., 2006; Zhang et al., 2009; Liao et al., 2019). In its original state, bee venom is a complex mixture of proteins (phospholipase A2, phospholipase B, and hyaluronidase), peptides (mainly melittin) and low-molecular-mass components such as histamine (Wehbe et al., 2019), while the bee-venom which was used in this study is composed of 99.9% melittin with removal of major toxic/allergic proteins. One previous study in Korea (using a diluted BV material without allergen-purification studied from 1998 to 2000) presented a 4-fold higher anaphylaxis incidence rate (0.03% of 32,000 treatments) compared to the present results (Hwang and Lee, 2000). Females are known to be more susceptible to ADRs, including anaphylaxis (Jensen-Jarolim and Untersmayr, 2008). In our results, the incidence rate of anaphylaxis (but not total hypersensitivity) was 3-fold higher in females than in males, as shown in the above MRI contrast agents (1.5-fold) (Jung et al., 2012a) and BVA (2.4-fold in Kim’s study, 2.7-fold in Hwang’s study) (Hwang and Lee, 2000; Kim et al., 2016). Previous clinical studies have reported a positive relationship between immune disorders and hypersensitivity reactions, likely a 1.7-fold higher incidence of adverse reactions to NSAIDs in subjects with autoimmune diseases (Blumenthal et al., 2017). Our study found that 40% of cases (6 out of 15 with hypersensitivity reactions) had underlying allergic diseases, but we cannot identify the exact correlation due to the lack of information for all subjects underwent BVA treatment. As in other studies, age did not affect the incidence of hypersensitivity or anaphylactic reactions in our study. Regarding the main symptoms of hypersensitivity reactions and their frequency, skin symptoms such as generalized itchiness and urticaria were the most common (12 cases, 80%) and disappeared within one or two days.

The subjects with a higher grade of severity (2 or 3 grade) complained of respiratory, gastrointestinal, cardiovascular, and neurological symptoms such as dyspnea, nausea, vomiting, abdominal pain, hypotension, or loss of consciousness, respectively. Although one patient was hospitalized for three days, and weakness and anxiety in two subjects were likely to last up to 30 days, all patients completely recovered (**Table 3**). The two severe cases with hypotension and loss of consciousness were treated with epinephrine and dexamethasone, while 13 cases (mild and moderate) were treated with antihistamine only or as a combination with dexamethasone according to international management guidelines for anaphylaxis. The average number of BVA treatments was 7.1 ± 14.8 in a total 8,580 subjects (**Table 1**), and 15 cases had the 6.9 ± 6.5 treatments (males 4.1 ± 3.4 and females 9.3 ± 7.9) before occurrence of the adverse reactions (**Table 3**, but the average number of treatments was not shown in table). Twelve cases of hypersensitivity reactions happened after several BVA treatments, while three cases showed it immediately on the first exposure in our hospital. However, these three subjects (P1, P5 and P6 in **Table 3**) had had BVA treatments in other clinics before visiting our hospital and two (P1 and P6) had experiences of mild allergic reactions (reconfirmed by phone call). In general, anaphylaxis is caused by IgE-mediated immunological release of chemical mediators; thus, repeated exposure to allergens can boost the production of IgE (Okano et al., 1999). One study reported that half of the bee-venom anaphylaxis-related deaths did not show allergic reactions to bee-venom exposure in the past (Golden, 2007). Interestingly, all anaphylaxis cases had long duration (between 129 ~ 391 days) between two time points, the last BVA treatment and the next previous BVA administration (**Table 3**). Currently, we cannot explain the reasons for our results; however, we have to pay attention to melittin itself, the core component of BV. Although the bee-venom used in present study was prepared by removing major allergens, melittin was known to induce minor allergic reaction (Jarisch et al., 1979). Melittin also can form a complex with phospholipase A2 (PLA2, the second most abundant compound), which consequentially cleaves cellular membrane phospholipids and then leads to inflammatory reaction (Mingarro et al., 1995). Accordingly, the repeated exposure of melittin of BVA could evoke the allergic reactions including anaphylactic shock in certain subjects. This might be supported by our result, which high grades (2 or 3) of hypersensitivity reactions occurred with greater number of BVA treatments (10.1 ± 6.7 times) compared to grade 1 (2.0 ± 1.3 times).

Skin tests are generally adapted for the prediction of a drug hypersensitivity (Brockow et al., 2002). At present, we recommend skin tests for every patient requiring BVA treatment, one before the first treatment and another after the 2-week interval period. However, it should be noted that all hypersensitive persons cannot be identified only through the skin test. High levels of serum IgE and tryptase are good indicators for type 1 hypersensitivity, including anaphylaxis (Rueff et al., 2009). In addition, the determination of the baseline serum tryptase levels in patients who underwent anaphylaxis to BVA could be helpful to rule out an unspecific mast cell activation disorder (Vitte, 2015). We however didn’t conduct those tests in present study. This would be the limitation of our present study along with a retrospective study.

Taken together, we can expect that the BVA-related risks of a hypersensitivity reaction and anaphylaxis are approximately 0.175 and 0.025% of subjects, and 0.025 and 0.007% of treatments. This risk of anaphylaxis shouldn’t be ignored, moreover requires to pay careful attention in clinical application of BVA due to the possibility of fatal outcome. This study would be a useful reference for the safe clinical application of bee-venom-derived interventions and their pharmaceutical development in the future.

## Data Availability Statement

The raw data supporting the conclusions of this article will be made available by the authors, without undue reservation, to any qualified researcher.

## Ethics Statement

The studies involving human participants were reviewed and approved by Institutional Review Board for Human Research in Dunsan Hospital of Daejeon University (approval number: DJDSKH-18-E-08-2). Written informed consent for participation was not provided by the participants’ legal guardians/next of kin because the institute proved the no-requirement of the written consent; however, we have obtained verbal consent from every subject identified as having a hypersensitivity reaction.

## Author Contributions

E-JL participated mainly in the design of the experiments and manuscript preparation. Y-CA, Y-CP, M-SO, and Y-IK contributed to the data collection and manuscript preparation including revision process. C-GS supervised whole processes of experiments and manuscript preparation. All authors contributed to the article and approved the submitted version.

## Funding

This study was supported by the Ministry of Education, Science and Technology (NRF-2018R1A6A1A03025221) and the Ministry of Health and Welfare of South Korea (grant number: HI15C0006).

## Conflict of Interest

The authors declare that the research was conducted in the absence of any commercial or financial relationships that could be construed as a potential conflict of interest.

## Abbreviations

ADR, Adverse drug reaction; BVA, Bee-venom acupuncture; EMR, Electronic medical record; ICD-10; International classification of diseases-10, NSAID, Nonsteroidal anti-inflammatory drug.
