# Supplementary material for: Incidence Rate of Hypersensitivity Reactions to Bee-Venom Acupuncture
Source: Front Pharmacol. 2020 Oct 7;11:545555. doi: 10.3389/fphar.2020.545555 (PMC7577189; doi:10.3389/fphar.2020.545555)
Supplement: Supplementary file 1 [file Table_1.pdf]

Supplementary table

Table S1. Severity of hypersensitivity reactions and criteria for anaphylaxis

Grading the severity by Brown SG	Signs and symptoms
Grade 1.<br>Mild (skin and subcutaneous tissues only)	Generalized erythema, urticaria, periorbital edema, or angioedema
Grade 2.<br>Moderate (features suggesting respiratory, cardiovascular, or gastrointestinal involvement)	Dyspnea, stridor, wheezing, nausea, vomiting, dizziness (presyncope), diaphoresis, chest or throat tightness, or abdominal pain
Grade 3.<br>Severe (hypoxia, hypotension, or neurological compromise)	Cyanosis or SpO_2 < 92% at any stage, hypotension (systolic blood pressure < 90 mmHg in adults), confusion, collapse, a loss of consciousness, or incontinence

Criteria for diagnosing anaphylaxis by the World Allergy Organization
Anaphylaxis is a highly likely event when any one of the following three criteria is fulfilled:<br>1. Acute onset of an illness (minutes to several hours) with the involvement of skin, mucosal tissue, or both. IT ALSO INVOLVES AT LEAST ONE OF THE FOLLOWING: A) respiratory distress, B) reduced blood pressure or associated symptoms of end-organ dysfunction OR<br>2. Two or more of the following that occurs rapidly when the patient is exposed to a likely allergen (minutes to several hours): A) the involvement of the skin-mucosal tissue, B) respiratory compromise (e.g., dyspnea, wheeze-bronchospasm, stridor, reduced PEF, hypoxemia), C) low blood pressure or associated symptoms, D) persistent gastrointestinal dysfunction OR<br>3. Lowered blood pressure of the patient after exposure to a known allergen (minutes to several hours). A) Infants and children: low systolic blood pressure (age-specific) or more than a 30% decrease in systolic blood pressure; B) Adults: systolic blood pressure of less than 90 mmHg or more than a 30% decrease from that person’s baseline
